# Early psychological intervention in accidentally injured children ages 2–16: a randomized controlled trial

**DOI:** 10.3402/ejpt.v5.24402

**Published:** 2014-06-27

**Authors:** Didier N. Kramer, Markus A. Landolt

**Affiliations:** 1 Department of Psychosomatics and Psychiatry, University Children's Hospital Zurich, Zurich, Switzerland; 2 Department of Child and Adolescent Health Psychology, Institute of Psychology, University of Zurich, Zurich, Switzerland

**Keywords:** Intervention outcome, mental health, posttraumatic stress, evidence-based practice

## Abstract

**Background:**

Road traffic accidents (RTA) and burns are frequent events in children. Although many children recover spontaneously, a considerable number develop long-term psychological sequelae. Evidence on early psychological interventions to prevent such long-term problems is still scarce for school-age children and completely lacking for pre-school children.

**Objectives:**

To evaluate the efficacy of an early two-session cognitive-behavioral intervention in 108 children ages 2–16 after RTAs and burns.

**Methods:**

Children assessed at risk for the development of posttraumatic stress disorder (PTSD) were randomly assigned to either a control group offered treatment as usual or an intervention group. Primary outcomes were PTSD, behavioral problems, and depression symptoms. Baseline and blinded 3- and 6-month follow-up assessments were conducted.

**Results:**

In pre-school children, no intervention effects were found. School-age children in the intervention group exhibited significantly fewer internalizing problems at 3-month follow-up relative to controls and a borderline significant time-by-group effect for PTSD intrusion symptoms was found (*p=*0.06).

**Conclusions:**

This is the first study examining the efficacy of an indicated, early psychological intervention among both school-age and pre-school-age children. Because the intervention was ineffective for young children, no evidence-based practice can currently be suggested. Given that parents of pre-school children perceived the intervention as helpful, brief counseling of parents in terms of psychoeducation and training in coping skills still should be provided by clinicians, despite the current lack of evidence. To prevent trauma-related disorders in school-age children, the intervention might be used in a step-wise manner, where only children at risk for long-term psychological maladjustment are provided with psychological support.

Road traffic accidents (RTA) and burns are frequent events in children. In 2011, roughly 19 and 28% of all non-fatal RTAs (4,266,777) and burns (418,239) in the US involved children (National Center for Injury Prevention and Control, 2011). In addition to the physical threat, children may also be psychologically traumatized after unintentional injuries. For instance, after an RTA, 10% of pre-school children (Meiser-Stedman, Smith, Glucksman, Yule, & Dalgleish, 2008) and 13% of school-age children (Olofsson, Bunketorp, & Andersson, 2009) suffer from posttraumatic stress disorder (PTSD). In another study, PTSD prevalence after burn injuries affected 13% of pre-school children (Graf, Schiestl, & Landolt, 2011) and almost 19% of school-age children (Landolt, Buehlmann, Maag, & Schiestl, 2009). Besides PTSD, children may also suffer from other persistent psychological problems after RTAs and burns, such as emotional and behavioral problems (Bakker, Maertens, Van Son, & Van Loey, 2013; Gillies, Barton, & Di Gallo, 2003). Fortunately, spontaneous recovery in children is common (De Young, Kenardy, Cobham, & Kimble, 2012). Consequently, not all injured children need psychological support after acute trauma. Targeted preventative care is therefore a reasonable approach that is also time- and cost-effective.

To successfully provide targeted care, reliable and valid screening instruments with good predictive values are required, such as the Child Trauma Screening Questionnaire (CTSQ; Kenardy, Spence, & Macleod, 2006) and Pediatric Emotional Distress Scale–Early Screener (PEDS-ES; Kramer, Hertli, & Landolt, 2013).

A recent meta-analysis examined the characteristics and efficacy of early psychological interventions in children after single trauma (Kramer & Landolt, 2011). The interventions had to be carried out within 4 weeks post-accident to be considered for inclusion. Seven studies from 1992 to 2011 were ultimately included, of which four were randomized controlled trials (RCT). Notably, Stallard et al., (2006) were the first who conducted an RCT on the effectiveness of psychological debriefing in children. Since publication of the meta-analysis by Kramer and Landolt, one further RCT has been published (Kassam-Adams et al., 2011). Methodological quality has varied greatly between studies. Sample sizes ranged from 24 to 158 children ages 7–18. Trauma types were very heterogeneous (e.g., unintentional injuries, physical or sexual abuse, or classmate's suicide). Merging the samples of all included studies, the number of PTSD diagnoses did not differ significantly between intervention and control condition (Kramer & Landolt, 2011). With respect to the single studies, only the intervention of Berkowitz, Stover, and Marans (2011) could reduce the rate of PTSD diagnoses. The meta-analysis revealed beneficial mean effect sizes of early interventions in school-age children for dissociation, anxiety, and arousal. However, considering the included studies separately, results were very heterogeneous with some studies finding no intervention effects at all. Despite these inconsistencies, the following components of an intervention were deemed important: psychoeducation, training of individual coping skills, presence of at least one parent, and most probably trauma narration. Previous research also suggests that multiple and age-adjusted sessions within the framework of a step-wise protocol where only children at high risk for long-term psychological maladjustment are provided with psychological support should be provided to children at risk (Kramer & Landolt, 2011). As stated earlier, methodological quality of the included studies varied widely, with only four of the included studies a randomized controlled trial. Based on this small number of high-quality studies, no final conclusions regarding the effectiveness of early interventions could be made. The authors suggested that further, methodologically sound RCTs on early psychological interventions in children were required (Kramer & Landolt, 2011). Moreover, although many pre-school-age children suffer unintentional injuries, no studies on early psychological interventions are available for this age group, meaning that intervention studies involving the young remain desperately needed.

The objective of this RCT was to examine the efficacy of a manualized and age-adjusted two-session early psychological intervention, both for pre-school and school children, ages 2–6 and 7–16, respectively, after RTAs and burns. We hypothesized that children receiving the intervention would report fewer PTSD symptoms and behavioral problems 3 and 6 months post-injury, compared to children given standard medical treatment. Additionally, for school-age children in the intervention group, a significant decrease in depression symptoms was expected.

## Methods

### Participants

Children and adolescents were recruited if all of the following criteria were met: 1) age 2–16, 2) Swiss residence, 3) medical treatment (in- or outpatient) after an RTA or burn, 4) at least one German-speaking parent and fluency in German for children ages 7–16, 5) no severe head injury (Glasgow Coma Scale <9), and 6) no prior intellectual impairment (physician's rating).

**Fig. 1:**
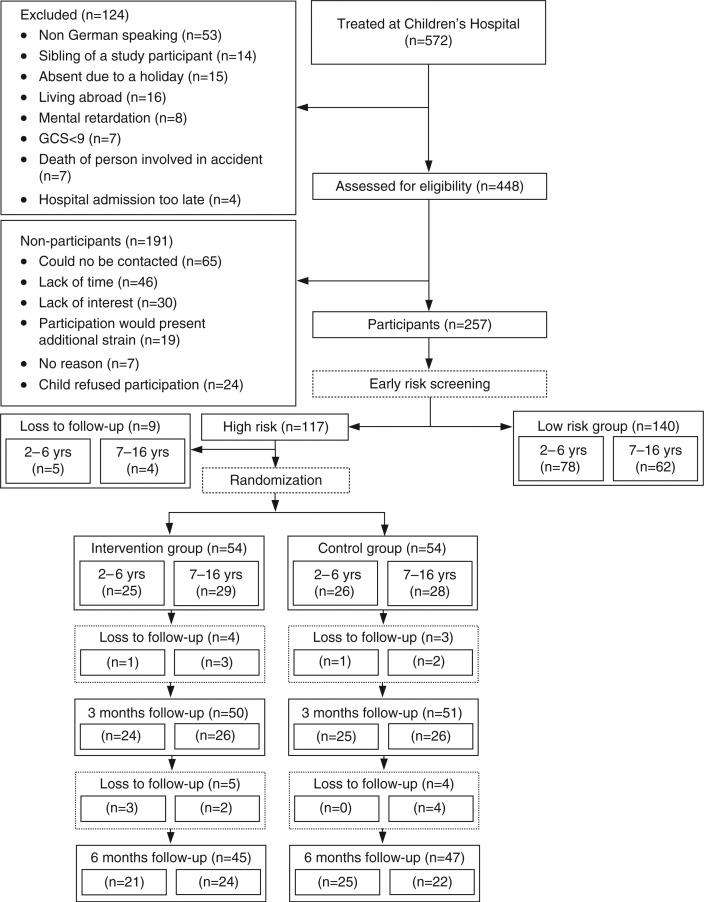
Participant flow chart.

Participant flow is illustrated in Fig. 1. Of the 572 children treated during the study period, 124 were excluded (Fig. 1). Another 191 could not be contacted or refused participation. Participants and non-participants did not differ in age (*t=*0.48, *p=*0.63), sex (*χ* ^2^ *=*0.28, *p=*0.60), type of accident (*χ* ^2^ *=*2.37, *p=*0.12), or type of medical treatment (inpatient vs. outpatient; *χ* ^2^ *=*2.04, *p=*0.15). Significantly more non-Swiss individuals declined participation (*χ* ^2^ *=*26.49, *p*<0.001). Participants had significantly longer hospital stays if treated as inpatients (*t=*−3.47, *p*<0.01) and were more severely injured (*t=*−3.00, *p*<0.01) than non-participants.

Of the 448 children assessed for eligibility, 257 children were screened for risk (response rate 57.4%). Of the children screened, 117 (45.5%) were allocated to the high-risk group. Seven children (6.0%) screened positive by surpassing the cutoff for the symptom-related measures (PEDS-ES or CTSQ); 80 (68.4%) screened positive because they had at least one additional risk factor present; and 30 (25.6%) screened positive for both criteria. Nine individuals dropped out of the study before randomization because they cancelled participation (*n=*1) or could not be contacted (*n=*8). The remaining 108 children were randomly assigned to either the control (*n=*54) or intervention group (*n=*54). Children in the control group received standard medical care, whereas children in the intervention group also received a two-session early intervention. All individuals in the intervention group completed both treatment sessions. Follow-up assessments with all data collected were completed for 47 (87.0%) and 45 (83.3%) children in the control and intervention group at 3 (T1) and 6 months (T2), respectively (Fig. 1). Of the 108 children randomized, 16 (14.8%) dropped out of the study: 9 (16.7%) from the intervention and 7 (13.0%) from the control condition.

### Procedures

The study was approved by the local ethics committee and enlisted as a registered RCT (NCT01085370). Via electronic hospital records, children were continuously recruited between May 2010 and September 2012 at University Children's Hospital in Switzerland. If a child met inclusion criteria, the family was approached within the first week of the child's accident. After written informed consent was obtained, screening was performed as soon as possible (either by phone or face-to-face). With families contacted by telephone, the parent's screening questionnaire was administered via an interview. In addition to the parent's screening questionnaire, children ages 7–16 were interviewed with a short screening measure of PTSD risk (either by phone or face-to-face). All screening interviews were carried out by the first author. The screening measures are described in the following section. Parents and school-age children completed the screening questionnaire within 5–19 days of the accident (Parents: *M=*8.72, SD=1.73 days, 86.4% within 10 days; Child: *M=*8.77, SD=2.01 days, 85.2% within 10 days). Children who screened positive were eligible for the RCT and a separate appointment was scheduled (at the hospital or family's home) for 10–16 days post-accident. During this appointment, with the child and at least one parent present, the standardized baseline assessment (T0) was performed. Either a parent (for 2- to 6-year-old children) or the child (7- to 16-year-old) was interviewed by the first author. Additional questionnaires were handed out to the parents to be completed and returned by mail after the session. Immediately following the baseline interview, a sealed envelope was opened to reveal random assignment to either the control or intervention group. The randomization list was stratified by child sex and age and generated by the software RANCODE 3.6 (IDV, Gauting, Germany). Directly after the baseline (T0) interview, the intervention was administered to those families randomized to receive the intervention. The second intervention session was completed 2 weeks later. Children randomized to the control group received standard medical care. The baseline interview and session 1 of the intervention took place approximately 2 weeks after the child's accident (*M=*13.74, SD=3.35 days), with session 2 roughly 2 weeks after session 1 (*M=*15.63, SD=5.33 days). T1 and T2 follow-up assessments were usually conducted approximately 3 and 6 months after the accident (T1: *M=*94.90, SD=13.23 days; T2: *M=*184.69, SD=13.23 days) in the family's home. The first author performed all recruitment, baseline interviews, and the intervention. Interviewers who conducted the follow-up interviews were Masters or Doctoral level students blinded to treatment arm. In return for participation, each child received 15 (low-risk group) or 40 (high-risk group) Euro after completing all assessments.

### Measures

#### Screening measures

To identify pre-school children at risk for persistent traumatic stress, the PEDS-ES (Kramer et al., 2013) was used. The PEDS-ES is a parent-reported instrument assessing the frequency of 21 reactive symptoms and behaviors rated on 4-point Likert scales (0–3). We used the PEDS’ original cutoff of >15 (Kramer et al., 2013). In the present sample, internal consistency of the scale was acceptable (*α=*.76). Additionally, parents were asked questions on further risk factors relating to pre-existing child behavioral problems; pre-existing chronic parental mental or physical illness; pre-traumatic life events in the family; parental feelings of guilt; and parental posttraumatic stress (Kramer et al., 2013). A pre-school child was considered to be at risk if either the PEDS’ original cutoff (>15) was surpassed or one of the additional risk factors was present.

For school-age children, the German version (TSK/10; Haas & Goldbeck, 2010) of the CTSQ (Kenardy et al., 2006) was administered. This measure assesses the presence of 10 PTSD symptoms (*yes*/*no*). Using a cutoff score of ≥5, good sensitivity (82%) and specificity (74%) for PTSD symptoms has been reported (Kenardy et al., 2006). Internal consistency in the present sample was *α=*.65, which is low but comparable to that reported by Kenardy et al. (2006). Additionally, each child was asked to rate his/her current distress with regards to guilt or life-threat during the accident on a 4-point Likert-scale (0–3). Parents were asked the same questions about additional risk factors as the parents of pre-school children (Kramer et al., 2013). The child was classified as at risk if either one risk factor was present, the CTSQ cutoff of ≥5 was surpassed, or one of the two additional questions asked to the child scored ≥2.

#### Acute and posttraumatic stress symptoms

In pre-school children, accident-related posttraumatic stress symptoms were assessed using the German version (Irblich & Hepton, 2006) of the *PTSD Semi-structured Interview and Observational Record for Infants and Young Children* (PTSDSSI; Scheeringa & Zeanah, 2005). The PTSDSSI assesses both the DSM-IV and alternative criteria for PTSD in pre-school children (Scheeringa, Zeanah, Myers, & Putnam, 2003). For the latter, five items (recollections, flashbacks, diminished interests, detachment, and irritability) were alternatively worded to ensure developmental sensitivity for young children (Scheeringa et al., 2003). Contrary to the DSM-IV algorithm, the alternative algorithm requires only one avoidance/numbing criterion (Scheeringa et al., 2003). Psychometric properties were previously reported as good (Scheeringa et al., 2003). In the present sample, Cronbach's *α* was good at T0/T2 (*α=*.84) and acceptable at T1 (*α=*.77).

In school-age children, accident-related acute stress symptoms were assessed using the German version (CAB; Fruhe, Kultalahti, Rothlein, & Rosner, 2008) of the *Acute Stress Checklist for Children* (Kassam-Adams, 2006), which consists of 26 items assessing acute stress symptoms, rated on 3-point Likert-scales (0–2). The instrument assigns a diagnosis of Acute Stress Disorder (ASD) according to the DSM-IV. The CAB was conducted as a structured interview with the child. Internal consistency in the current sample was good (*α=*.87).

The German version (IPS-P-KJ; Steil & Füchsel, 2006) of the *Clinician-Administered PTSD Scale for Children and Adolescents* (CAPS-CA; Nader et al., 2002) was used to assess the diagnosis and symptoms of PTSD in school-age children according to DSM-IV criteria. Good psychometric properties were reported (Steil & Füchsel, 2006). Symptom frequency and intensity are scored on 5-point Likert-scales (0–4). Our Cronbach's *α* values were excellent (T1: *α=*.95; T2: *α=*.94).

Given that no instruments currently exist to assess pre-schooler ASD symptoms, the PTSDSSI was also used at T0. ASD/PTSD symptom severity scores were obtained by adding the scores of all items. A full ASD/PTSD diagnosis was based on the alternative algorithm for pre-school children (Scheeringa, Zeanah, Myers, & Putnam, 2005) and diagnosed according to the DSM-IV for school-age children. According to manual guidelines, for both the PTSDSSI and the CAB, an item was considered present when rated ≥1, and for the IBS-P-KJ when frequency was ≥1 and intensity ≥2.

#### Behavioral problems

Behavioral problems were assessed with the German versions of the *Child Behavior Checklist* (CBCL) for pre-school (CBCL 1½–5 (100 items); Arbeitsgruppe Deutsche Child Behavior Checklist, 2002) and school-age children (CBCL/4–18 (120 items); Steinhausen, Winkler Metzke, & Kannenberg, 1996), both parental proxy-report questionnaires with excellent psychometric properties (Achenbach & Rescorla, 2000; Steinhausen et al., 1996). Each item is coded on a 3-point Likert-scale (0–2). For the present study, the three broadband-scales for total, internalizing, and externalizing problems were used.

Because no Swiss or German reference data are available for pre-school children, US reference data were used (T-scores; Achenbach & Rescorla, 2000). In the present sample, internal consistency for the Total Scale was excellent (T0: *α=*.94; T1: *α=*.96; T2: *α=*.94), acceptable to excellent for the Internalizing Problems Scale (T0: *α=*.77; T1: *α=*.90; T2: *α=*.84), and good to excellent for the Externalizing Problems Scale (T0: *α=*.87; T1: *α=*.92; T2: *α=*.90).

For school-age children, raw data were transformed into T-scores, based on a Swiss community reference sample (Steinhausen et al., 1996). In the present study, internal consistency for the Total Scale was excellent (T0: *α=*.94; T1: *α=*.93; T2: *α=*.94), and good to excellent for internalizing (T0: *α=*.88; T1: *α=*.87; T2: *α=*.91) and externalizing problems (T0: *α=*.88; T1: *α=*.85; T2: *α=*.92).

#### Depression symptoms

The number of depression symptoms was assessed in school-age children via the German version (DIKJ; Stiensmeier-Pelster, 2000) of the *Children's Depression Inventory* (CDI; Kovacs, 1985). Each of the 26 items is scored on a 3-point Likert-scale (0–2). By summing these scores, a total score was generated. German reference data (T-scores) were used (Stiensmeier-Pelster, 2000). Good psychometric properties were reported (Stiensmeier-Pelster, 2011). In the present sample, Cronbach's *α* values were good to excellent (T0: *α=*.84; T1: *α=*.90; T2: *α=*.89).

#### Subjective intervention evaluation

To evaluate the participants’ perception of the intervention, mothers and school-age children were asked at 6-month follow-up whether or not they read the brochure. In addition, they were asked to rate the perceived helpfulness of the psychoeducation leaflet and the intervention on 5-point Likert-scales, ranging from *not helpful* (0) to *very helpful* (4). Ratings of perceived distress caused by the intervention were assessed on a 5-point Likert-scale, ranging from *not distressing* (0) to *very strongly distressing* (4).

#### Demographics and medical variables

Demographics were retrieved from hospital records. To compute socioeconomic status (SES), paternal occupation and maternal education were assessed on 6-point ordinal scales and summed. Using this score, parents were allocated to the lower (2–5), middle (6–9), or upper social class (10–12); this measure is a proven valid indicator of SES in Switzerland (Landolt, Vollrath, & Ribi, 2002). Injury severity was rated using the Modified Injury Severity Scale (Mayer, Matlak, Johnson, & Walker, 1980), ranging from 1 to 75, with higher scores indicating more severe injury.

### Standard medical care

Standard medical care, including clinical diagnostics and comprehensive medical treatment, was provided to all 572 children. Depending on the child's injury, staff members from different disciplines were available for treatment (surgeons, pediatricians, physical therapists, etc.). Although not routinely provided, psychological support also was available. In the present study, families of 10 pre-school and 17 school-age children received additional psychological support. The control and intervention groups did not differ in the number of psychological therapy sessions (Table 1) or type of psychological support (psychoeducation, *p=*0.39; training of coping strategies, *p=*1.00; exposure by trauma narrative, *p=*0.86).

**Table 1:** Comparison of demographic and medical characteristics between intervention and control groups (*N*=108)

	2–6 years						7–16 years					
		
	*M* (SD) or *N* (%)						*M* (SD) or *N* (%)					
						
Characteristics	Intervention Group		Control Group		*t* or *χ* ^2^	*p*	Intervention Group		Control Group		*t* or χ^2^	*p*
Sample size	25	(46.3)	26	(48.1)	–	–	29	(53.7)	28	(51.9)	–	–
Age (years)	4.10	(1.29)	4.44	(1.69)	0.81	0.43	11.00	(2.46)	11.01	(2.73)	0.02	0.99
Range	2.33–7.00		2.00–6.83				7.08–15.08		7.17–16.00			
Sex												
Male	15	(60.0)	15	(57.7)			21	(72.4)	20	(71.4)		
Female	10	(40.0)	11	(42.3)	0.03	0.87	8	(27.6)	8	(28.6)	0.01	0.93
Socioeconomic status												
Lower	2	(8.0)	2	(7.7)			0	(0.0)	0	(0.0)		
Middle	9	(36.0)	10	(38.5)			14	(48.3)	20	(71.4)		
Upper	7	(28.0)	9	(34.6)	0.07	0.97	14	(48.3)	5	(17.9)	5.17	0.02
Unknown	7	(28.0)	5	(19.2)			1	(3.4)	3	(10.7)		
Mean score (SD)	8.67	(2.45)	8.90	(2.68)	0.29	0.78	9.54	(1.60)	8.24	(1.45)	−3.08	0.03
Range	2.00–12.00		2.00–12.00				6.00–12.00		6.00–11.00			
RTA	9	(36.0)	8	(30.8)			18	(62.1)	22	(78.6)		
Burn	16	(64.0)	18	(69.2)	0.16	0.69	11	(37.9)	6	(21.4)	1.85	0.17
Medical treatment												
Inpatient	11	(44.0)	9	(34.6)			17	(58.6)	18	(64.3)		
Outpatient only	14	(56.0)	17	(65.4)	0.47	0.49	12	(41.4)	10	(35.7)	0.19	0.66
Additional psychological support within standard care												
No	20	(80.0)	21	(80.8)			20	(69.0)	20	(71.4)		
Yes	5	(20.0)	5	(19.2)	–a	0.61	9	(31.0)	8	(28.6)	0.04	0.84
Days of hospital stay (inpatients only)	8.64	(9.14)	7.56	(7.94)	–2.79	0.78	18.65	(25.31)	10.44	(12.73)	–1.20	0.24
Range	1.00–27.00		1.00–21.00				1.00–74.00		1.00–54.00			
Injury Severity Score	4.20	(5.93)	2.19	(3.41)	−1.49	0.14	7.24	(9.68)	5.64	(6.87)	−0.72	0.48
Range	0.00–29.00		0.00–18.00				0.00–45.00		1.00–34.00			

*Note*: RTA=road traffic accident.

a Fisher's exact test was used.

### Early psychological intervention for children and parents

Early psychological intervention for children and parents (EPICAP) is a further development of the cognitive-behavioral intervention evaluated by Zehnder, Meuli, and Landolt (2010). They provided a single-session intervention to 7- to 16-year-old children after a road traffic accident following a structured, 4-step process. First, the accident was reconstructed in detail by means of drawings and accident-related toys. Second, dysfunctional accident-related appraisals were identified and the child was supported in modifying them. Third, psychoeducation on common acute stress reaction was provided to normalize the child's stress symptoms, and coping skills for dealing with these reactions were discussed. Fourth, a leaflet was handed out containing written information on posttraumatic stress and a contact address. Drawing on their results and the meta-analysis by Kramer and Landolt (2011), the initial intervention was modified in three ways: 1) patients participated in two sessions instead of one to spend more time on individual coping strategies; 2) the trauma narrative was age-adjusted; and 3) an intervention manual for pre-school children was created. Because this is the first study on early interventions in pre-school children, children ages 2–6 received the same intervention irrespective of their current cognitive developmental stage. To ensure that the youngest participants also could profit from the intervention, the parents of these young children were primarily addressed. The concept of three components was maintained. In component 1, detailed reconstruction of the accident was performed: children ages 2–11 reconstructed the accident using toy figures, and adolescents utilized less-childlike items (e.g., small model cars and simple wooden figures). Although children ages ≤6 were encouraged to retell the accident by themselves, some needed to be supported by their caregiver (i.e., the caregiver led the reconstruction while the child watched). Previous findings suggest that incomplete trauma memory has a large impact on the initial development of PTSD (Stallard & Smith, 2007). Consequently, construction of a trauma narrative might be essential in the early aftermath of a traumatic event. Accordingly, trauma reconstruction in the EPICAP intervention was aimed at constructing a complete (explicit) trauma memory. Moreover, children and parents were intended to be exposed to the trauma during trauma reconstruction.

In component 2, during session 1, psychoeducation on child acute stress reactions and *general* age-appropriate coping strategies (e.g., talking about the accident or reestablishing daily routines in the child's life) were provided orally and in written form to parents and school-age children (leaflet). This information aimed to normalize posttraumatic stress reactions and help the child to cope with symptoms. For pre-school children, parents were instructed on how to cope with their child's stress reactions.

During component 3, age-appropriate and standardized coping skills were practiced with school-age children for each of their current PTSD symptoms (e.g., relaxation skills or exposure strategies). To help them cope with their child's current PTSD symptoms, appropriate strategies were discussed with parents of pre-school children (e.g., how to react to sleeping problems and temper tantrums).

Components 1 and 2 were part of session 1, whereas component 3 was provided in session 2. During the intervention, at least one parent had to be present. The EPICAP-manual is available upon request. All intervention sessions were provided by the first author and supervised by the last author. Hence, the procedure was identical for all individuals in the intervention group.

### Statistical analyses

Data were analyzed using SPSS 20 (SPSS Inc., Chicago, IL). First, descriptive analyses for demographics and drop-out analyses were conducted. All analyses were performed with two-sided tests and a *p*-value <0.05 was considered significant. Nominal variables were analyzed using the *χ* ^2^-test or Fisher's exact test. For continuous data, Student's *t-*tests were used. Statistical analyses were conducted for the pre-school- and school-age children separately because different outcome measures with incommensurable scales were administered.

PTSD symptom severity and PTSD diagnosis (*yes*/*no*) were the primary outcomes of interest. Secondary outcomes were child internalizing and externalizing problems (for ages 2–16) and depression symptoms (for ages 7–16). Descriptive statistics for these variables are presented in Tables 2 and 3. Pre- to post-treatment changes in primary and secondary outcomes were analyzed using univariate repeated-measures analyses of covariance (ANCOVAs). Baseline scores for the dependent variables were included as covariates. Time-by-group interactions and *post-hoc* Student's *t*-tests were used for all follow-up time points to indicate whether the change over time was different between groups. Only those children who had valid data at all three assessment time points were included in analysis. Missing data were not imputed. This led to slightly different sample sizes for different analyses. For each analysis, actual sample sizes are reported in Tables 4 and 5. *Standard mean differences* (SMD) were computed based upon the marginal means and standard errors estimated by ANCOVA. Negative SMDs indicate that the intervention group was superior to controls. The magnitude of the SMD was interpreted by means of Cohen's (1988) categories: 0.2–0.5 (small effect); 0.5–0.8 (medium effect); and >0.8 (large effect).

**Table 2:** Descriptives of baseline, 3- and 6-month follow-up for intervention and control condition in 2- to 6-year-old children (*N*=51)

	Intervention group									Control group								
		
	Baseline			3 months			6 months			Baseline			3 months			6 months		
						
	*N*	*M*	SD	*N*	*M*	SD	*N*	*M*	SD	*N*	*M*	SD	*N*	*M*	SD	*N*	*M*	SD
ASD/PTSD																		
Total symptom severity	25	6.76	5.64	24	5.33	5.30	21	4.33	6.67	26	4.92	3.42	25	4.64	4.51	25	2.84	2.67
Intrusion symptom severity	25	3.04	2.39	24	2.83	2.65	21	1.90	2.51	26	1.96	1.59	25	2.28	2.28	25	1.52	1.83
Avoidance symptom severity	25	1.88	1.99	24	1.21	1.67	21	1.00	2.39	26	1.38	1.24	25	1.00	1.12	25	0.52	1.00
Arousal symptom severity	25	1.84	2.06	24	1.29	1.65	21	1.43	2.27	26	1.58	1.60	25	1.36	1.89	25	0.80	1.00
Behavior problems																		
Total score (T-score)	17	44.12	8.74	18	43.50	12.30	17	43.65	8.48	22	42.73	11.22	24	41.58	11.28	22	39.50	10.60
Internalizing score (T-score)	18	44.72	9.80	18	45.28	12.19	17	44.47	8.17	22	43.27	11.24	24	43.08	11.26	22	39.91	11.98
Externalizing score (T-score)	18	45.22	8.22	18	43.44	11.80	17	45.00	8.65	22	44.64	10.20	24	43.00	10.68	22	40.91	9.94

**Table 3:** Descriptives of baseline, 3- and 6-month follow-up for intervention and control condition in 7- to 16-year-old children (*N*=57)

	Intervention group									Control group								
		
	Baseline			3 months			6 months			Baseline			3 months			6 months		
						
	*N*	*M*	SD	*N*	*M*	SD	*N*	*M*	SD	*N*	*M*	SD	*N*	*M*	SD	*N*	*M*	SD
ASD/PTSD^a^																		
Total symptom severity	29	10.10	7.46	26	17.42	26.02	24	11.13	16.26	28	10.89	7.08	25	17.76	19.68	21	11.57	14.24
Intrusion symptom severity	29	2.83	2.47	26	4.96	8.38	24	3.33	6.59	28	2.61	2.20	25	6.04	7.69	21	3.10	4.97
Avoidance symptom severity	29	2.45	2.37	26	6.15	10.20	24	3.96	6.46	28	3.07	2.57	25	6.32	7.41	22	4.82	5.65
Arousal symptom severity	29	2.83	2.58	26	6.31	9.56	24	3.83	5.56	28	2.64	2.47	25	5.40	7.07	21	3.43	4.85
Behavior Problems																		
Total score (T-score)	28	50.43	11.94	26	48.00	11.70	23	50.26	11.27	26	51.69	11.56	21	50.71	8.88	22	49.64	10.75
Internalizing score (T-score)	27	52.07	11.37	25	47.04	10.82	21	47.52	11.36	26	50.23	9.05	22	50.73	8.77	22	47.45	10.41
Externalizing score (T-score)	28	48.82	11.23	26	48.69	11.86	23	50.70	12.18	26	50.62	11.33	22	50.59	8.49	22	50.27	12.36
Depression symptoms (T-score)	29	48.66	9.95	26	45.42	11.39	24	43.58	9.73	28	43.04	7.24	24	43.08	9.63	22	40.86	8.63

a ASD measure used at baseline has a different scaling than the PTSD measure used at 3- and 6-months follow-up.

**Table 4:** Comparison of primary and secondary outcome variables between intervention and control conditions in 2- to 6-year-old children (*N*=51)

							ANCOVA						
							
	Intervention group			Control group			Time×group		Time		Post hoc test		
					
	*N*	EMM	(SE)	*N*	EMM	(SE)	*F*^a^	*p*	*F*^a^	*p*	*t*	*p*	SMD
PTSD													
Total symptom severity													
3 months	21	4.98	0.86	25	5.26	0.79					0.24	0.81	−0.07
6 months	21	3.57	0.81	25	3.48	0.74	0.12	0.73	4.55	0.04	−0.08	0.94	0.02
Intrusion symptom severity													
3 months	21	2.66	0.47	25	2.60	0.43					−0.09	0.93	0.03
6 months	21	1.51	0.38	25	1.85	0.34	0.35	0.56	3.72	0.06	0.64	0.52	−0.19
Avoidance symptom severity													
3 months	21	1.08	0.27	25	1.14	0.24					0.17	0.87	−0.05
6 months	21	0.81	0.33	25	0.68	0.30	0.26	0.62	4.00	0.05	−0.27	0.79	0.08
Arousal symptom severity													
3 months	21	1.35	0.36	25	1.42	0.33					0.15	0.88	−0.04
6 months	21	1.35	0.34	25	0.86	0.31	1.24	0.27	0.54	0.47	−1.08	0.29	0.31
Behavior problems													
Total score (T-score)													
3 months	12	42.11	2.34	20	39.53	1.81					−0.87	0.39	0.30
6 months	12	41.87	2.44	20	37.68	1.89	0.43	0.52	1.53	0.23	−1.35	0.19	0.47
Internalizing score (T-score)													
3 months	13	42.10	2.22	20	41.09	1.79					−0.36	0.72	0.12
6 months	13	42.51	2.49	20	37.92	2.01	1.23	0.28	0.18	0.68	−1.44	0.16	0.48
Externalizing score (T-score)													
3 months	13	44.17	2.30	20	41.44	1.85					−0.92	0.36	0.31
6 months	13	43.42	2.22	20	39.58	1.79	0.20	0.66	1.02	0.32	−1.35	0.19	0.45

*Note*: EMM=estimated marginal mean; SE=standard error; SMD=standard mean difference.

a df=1.

## Results

### Sample characteristics and the baseline assessment

For children ages 2–6, no significant demographic or medical characteristic differences between the two study groups were observed (Table 1). In school-age children, the intervention group included more families of higher SES (Table 1); consequently, SES was included as an additional covariate in the analysis of school-age children. Symptom levels at baseline differed significantly only in school-age children, with the intervention group exhibiting more depressive symptoms than controls (*t=*−2.089; *p*<0.05).

Comparing drop-outs and those completing the study revealed no significant differences in demographic or medical characteristic variables, or in baseline symptoms, even when pre-school- and school-age children were analyzed separately (data not shown). Therefore, despite the relatively high attrition rate (14.8%), no selection bias was evident.

### Efficacy of the EPICAP intervention

Tables 4 and 5 compare primary and secondary outcomes between the two treatment groups in pre-school- and school-age children, respectively. In pre-school children, a significant decrease over time was identified with regards to total PTSD symptom severity. However, no significant time-by-group interactions or *post hoc t*-tests were identified with regards to PTSD symptom severity or behavioral problems (Table 4).

Among school-age children, a significant decrease over time was discovered for depressive symptoms (Table 5); however, there again was no significant time-by-group interaction. The time-by-group interaction was almost significant for intrusion symptom severity (*p=*0.06) with a small negative effect size at T1 (SMD=−0.50). With regards to internalizing problems, a significant group difference with a large effect size was found at T1 (SMD=−1.11), whereas the difference at T2 was still of medium magnitude, but non-significant (SMD=−0.53). In sum, intervention effects were more pronounced at T1 than at T2.

**Table 5:** Comparison of primary and secondary outcome variables between intervention and control conditions in 7- to 16-year-old children (*N*=57)

							ANCOVA						
							
	Intervention group			Control group			Time×group		Time		Post hoc test		
					
	*N*	EMM	(SE)	*N*	EMM	(SE)	*F*^a^	*p*	*F*^a^	*p*	*t*	*p*	SMD
PTSD													
Total symptom severity													
3 months	24	17.38	4.39	20	21.29	4.85					0.57	0.57	−0.17
6 months	24	11.52	3.04	20	10.58	3.36	0.72	0.40	2.46	0.13	−0.20	0.84	0.06
Intrusion symptom severity													
3 months	24	4.20	1.63	20	8.36	1.80					1.63	0.11	−0.50
6 months	24	3.19	1.26	20	3.17	1.39	3.85	0.06	2.82	0.10	−0.01	0.99	0.00
Avoidance symptom severity													
3 months	24	6.44	1.86	21	7.02	2.00					0.20	0.84	−0.06
6 months	24	4.14	1.31	21	4.55	1.41	0.01	0.93	1.41	0.24	0.20	0.84	−0.06
Arousal symptom severity													
3 months	24	6.54	1.56	20	5.80	1.72					−0.31	0.76	0.09
6 months	24	4.11	0.95	20	2.72	1.05	0.07	0.79	0.96	0.33	−0.94	0.35	0.29
Behavior problems													
Total score (T-score)													
3 months	23	48.40	1.41	19	51.36	1.57					1.36	0.18	−0.42
6 months	23	49.73	1.40	19	49.01	1.55	2.11	0.15	0.43	0.52	−0.33	0.74	0.10
Internalizing score (T-score)													
3 months	20	45.73	1.43	20	53.17	1.43					3.44	0.00	−1.11
6 months	20	45.37	1.73	20	49.68	1.73	1.51	0.23	1.52	0.23	1.65	0.11	−0.53
Externalizing score (T-score)													
3 months	23	49.31	1.42	20	49.99	1.53					0.31	0.76	−0.10
6 months	23	50.80	1.97	20	50.48	2.12	0.13	0.72	0.27	0.60	−0.11	0.92	0.03
Depression symptoms													
Total T-score													
3 months	24	44.28	1.87	20	45.02	2.07					0.25	0.81	−0.08
6 months	24	43.15	1.67	20	41.32	1.85	1.36	0.25	4.85	0.03	−0.69	0.49	0.21

*Note*: EMM=estimated marginal mean; SE=standard error; SMD=standard mean difference.

a df=1.

No intervention effects were evident with regards to the diagnosis of ASD/PTSD (Table 6). Across conditions, among pre-school children, the number of diagnoses decreased over time from 21.6% at T0 to 7.1% at T2. In school-age children, the prevalence remained almost stable over time with only two (3.5%), three (5.9%), and two children (4.4%) meeting criteria for ASD at T0 and PTSD at T1 and T2, respectively. Notably, in both age-groups, no ASD/PTSD diagnoses were found within the low-risk group at either baseline or T2.

**Table 6:** Comparison of PTSD diagnoses by age group and intervention condition

	2–6 years								7–16 years							
		
	Intervention group			Control group			Statistics		Intervention group			Control group			Statistics	
						
	*N*	*n* with PTSD	%	*N*	*n* with PTSD	%	*χ* ^2^	*p*	*N*	*n* with PTSD	%	*N*	*n* with PTSD	%	*χ* ^2^	*p*
ASD at baseline	25	6	24.0	26	5	19.2	0.171	0.68	29	1	3.4	28	1	3.6	–a	1.00
PTSD at 3 months	24	2	8.3	25	3	12.0	–a	1.00	26	2	7.7	25	1	4.0	–a	1.00
PTSD at 6 months	20	3	15.0	22	0	0.0	–a	0.10	24	1	4.2	21	1	4.8	–a	1.00

a Fisher's exact test was used.

### Subjective evaluation of the intervention

In our study, the vast majority of mothers studied the psychoeducation leaflet (78.6%), whereas only a few children ages 7–16 looked at the brochure (36.4%). Most mothers indicated that they found both the intervention (68.8%) and the information leaflet (69.2%) for their 2- to 6-year-old child *helpful* or *very helpful*. Conversely, only a few mothers of children ages 7–16 indicated that they found the intervention (38.5%) or the information leaflet (8.3%) *helpful* or *very helpful*. Only 3.6% of the mothers and 9.1% of the school-age children found the intervention *strongly* or *very strongly distressing*.

## Discussion

The current RCT is the first to assess the efficacy of an age-adjusted early psychological intervention in a sample of injured children ages 2–16. Contrary to our hypothesis, the intervention failed to produce any effect on PTSD symptom severity, the rate of PTSD diagnoses, or behavioral problems in preschoolers. Among 7- to 16-year-old children, however, our findings tentatively support a beneficial intervention effect: children receiving the intervention had borderline less intrusion PTSD symptom severity (*p=*0.06) and significantly fewer internalizing problems with a small (SMD=−0.50) and large (SMD=−1.11) effect size, respectively, at 3-month follow-up. Thereby, effect sizes were comparable to those reported for previous RCTs (Kramer & Landolt, 2011). Given that differences at 3 months were more pronounced than at 6 months, the intervention might have helped the children to recover more quickly. Interestingly, no beneficial effects were noted for externalizing problems, potentially due to the intervention's focus on trauma reconstruction, which might have helped the child to create a better-integrated trauma memory (Neuner et al., 2008). Possibly, externalizing problems might be better addressed by training children in specific coping strategies or via educational counseling for parents. Although session 2 addressed the former, it seems that children might not have been able to transfer this knowledge into daily life. Consequently, either *in-vivo* exposure and/or further sessions to deepen and monitor competency in coping strategies might have been necessary.

ASD/PTSD diagnosis rates were low in school-age children. This could be the effect of enhanced trauma-informed care over the past decade at University Children's Hospital Zurich in Switzerland. Consistent with previous findings (Kramer & Landolt, 2011), the intervention was ineffective at reducing PTSD diagnosis rates.

Though included among internalizing problems, depressive symptoms were not affected by the intervention. Similarly, Zehnder et al. (2010) failed to identify any interventional effects on depressive symptoms in 12- to 16-year-old children, but did so in those ages 7–11. Unfortunately, our small sample size did not allow for subgroup analysis by age. However, it should be noted that the findings of previous RCTs are mostly heterogeneous (Kramer & Landolt, 2011). Only one study revealed beneficial effects across all outcome variables (Berkowitz et al., 2011). Others identified no intervention effects at all (Kassam-Adams et al., 2011; Kramer & Landolt, 2011) or reported inconsistent findings across different outcome variables (Kramer & Landolt, 2011). One interpretation of these inconsistencies is that the types of intervention differed between studies. Furthermore, most RCTs involved small subject samples, and the interventions and analyses were not age specific. Further research should include larger samples stratified by age.

The results of the subjective ratings show that mothers of young children found the intervention helpful and not distressing, whereas neither school-age children nor their parents found the intervention helpful or distressing. One possible explanation for this surprising result in school-age children could be that, because this group was only slightly symptomatic, they could not benefit from the intervention and therefore did not perceive it as helpful. One might additionally wonder whether motivation to participate might have had an impact on perceived helpfulness. To empirically examine this relationship, the wish to receive early help should be assessed during the baseline assessment, which we did not do.

### Limitations

This study has several limitations. First, the participation rate was low (57.4%), limiting the results’ generalizability; however, response rates in previous RCTs on early psychological interventions were similarly low (Kassam-Adams et al., 2011; Zehnder et al., 2010), though this does not reduce the importance of this limitation. The present findings also must be extrapolated with caution to foreign immigrants as well as to individuals who tend to have shorter hospital stays and be less severely injured, because these characteristics differed significantly between our participants and non-participants. The representativeness of the results may therefore be jeopardized in this regard. Unfortunately, we do not know how the results would be without these differences and with a higher participation rate. Second, sample sizes were small and the degree of morbidity we observed was low. Consequently, statistical analyses lacked power and significant intervention effects were harder to identify. Results should therefore be considered tentative. Third, children in the control condition were interviewed at baseline. This might have had a beneficial effect by acknowledging, validating, and normalizing the child's symptoms (Stallard et al., 2006). Including two control groups – one with and the other without any baseline assessment – could remedy this problem by testing whether a baseline assessment itself influences the level and number of later stress symptoms. Despite these limitations, the present study has several strengths, including its randomized-controlled prospective design with two blinded follow-up assessments, where highly standardized instruments were used. Moreover, the study followed a step-wise protocol, providing manualized two-session intervention only to children screened at risk for long-term psychological maladjustment.

### Implications for clinicians and researchers

The EPICAP intervention was ineffective in pre-school children. Considering that there have been no other studies on early interventions in this age group, currently no evidence-based approach can be suggested for children younger than 6. Because parents of pre-school children perceived the intervention as helpful, clinicians still might provide psychoeducation and training in coping skills for a child's or parent's acute stress symptoms, despite today's lack of evidence. This includes, for instance, clinical advice that parents should talk openly about the accident with their child. Such educational guidelines could be especially helpful to parents during the highly distressing acute phase after their child's accident, empowering them with goal-oriented activities they themselves can undertake to enhance their child's and their own health.

Although no significant intervention effects were discovered, some lessons can be learned from this study with regards to future early intervention studies with pre-school children. First, because the time spent on the trauma narrative was restricted, trauma reconstruction primarily focused on the traumatic event, while subsequent medical procedures were addressed only marginally. Because medical procedures significantly affect a child's PTSD symptoms (Graf et al., 2011), early intervention should also address these stressors. Second, one could argue that, among young children, brief trauma reconstructions might be too abstract, such that these children might benefit more from *in-vivo* exposures. For example, parents can help their children to carefully confront trauma reminders or triggers, such as the place where the accident happened. Third, research on risk factors for PTSD symptoms has demonstrated the importance of parental factors (De Young, Kenardy, & Cobham, 2011). Although we included parents in the intervention and provided coping skills on child PTSD symptoms, relational aspects might have been missed. Scheeringa and Zeanah (2001) suggested that parental re-enactment, withdrawal/unavailability, and overprotection all negatively influence a young child's recovery from PTSD. Hence, any adaptation of the EPICAP intervention for pre-school children should integrate parenting strategies and parental distress.

There is tentative evidence that school-age children who received the two-session EPICAP intervention recovered more quickly from intrusive PTSD symptoms and from internalizing problems. Consistent with previously published findings (Kramer & Landolt, 2011), we therefore suggest to follow a step-wise protocol, providing early interventions only to children at risk. Interventions should involve at least one parent, provide psychoeducation, and teach individual coping skills. Whether including a trauma narrative contributes to better recovery remains unclear; however, because no deleterious effects were found, providing some sort of trauma exposure among children at risk might be appropriate.
